# Papillary cystadenoma of the epididymis

**DOI:** 10.4322/acr.2021.374

**Published:** 2022-04-14

**Authors:** Olga Lopez, Hisham F. Bahmad, Ruben Delgado, Billy H. Cordon, Robert Poppiti, Lydia Howard

**Affiliations:** 1 Florida International University, Herbert Wertheim College of Medicine, Miami, FL, USA; 2 Mount Sinai Medical Center, Arkadi M. Rywlin M.D. Department of Pathology and Laboratory Medicine, Miami Beach, FL, USA; 3 Mount Sinai Medical Center, Division of Urology, Miami Beach, FL, USA

**Keywords:** Case Reports, Epididymis, Cystadenoma, Papillary, von Hippel-Lindau Disease

## Abstract

**Background:**

Papillary cystadenoma is a rare benign neoplasm of the epididymis. It may occur sporadically or in association with von Hippel-Lindau disease (VHLD). Papillary cystadenoma of the epididymis (PCE) is a benign mimic of metastatic clear cell renal cell carcinoma (CCRCC) given their histologic similarities.

**Case presentation:**

Herein, we present the case of a 40-year-old man with a four-year history of microhematuria and a recently detected right paratesticular mass. A testicular sonogram revealed a hypoechoic, hypervascular solid mass in the right epididymal head treated by surgical excision. Histopathological examination demonstrated a 1.1 cm papillary cystadenoma of the epididymis. Genetic testing performed later showed no signs of VHLD. However, heterozygous mutations in three genes - *CASR*, *POT1*, and *RAD51D* - were found which have never been reported in PCE before.

**Conclusions:**

Papillary cystadenoma of the epididymis should always be considered in the differential diagnosis of epididymal lesions, especially those that are cystic. The mainstay of treatment remains surgical excision, which provides an excellent prognosis.

## INTRODUCTION

Papillary cystadenoma is a rare benign epithelial neoplasm of the epididymis, derived from the mesonephros, and characterized by a prominent papillary architecture.^1^ It can occur sporadically or in association with von Hippel-Lindau disease (VHLD). In fact, there is a strong association with VHLD; wherein more than one-third of papillary cystadenomas of the epididymis (PCE) reported in the literature have occurred in patients with VHLD, particularly when bilateral tumors are present.^1^ PCE is the second most common neoplasm of the epididymis after adenomatoid tumor.^2^^,^^3^ Other benign epididymal neoplasms include leiomyoma, serous (nonpapillary) cystadenoma, cavernous hemangioma, and melanotic neuroectodermal tumor, whereas malignant epididymal tumors include adenocarcinoma, mesothelioma, and metastatic clear cell renal cell carcinoma (CCRCC).^1^

The tumor, we report, was first described in 1956 by Sherrick^4^ in a 21-year-old patient. However, historically, Brandt^5^ referred to this entity in 1921 as an “epididymal cyst” found at autopsy of a patient with VHLD. Since that time, approximately 70 reports of this neoplasm have been published in the English-language literature.^1^^,^^2^^,^^4^^-^33 Given similar histology consisting of clear cells, PCE may be mistaken for metastatic CCRCC, clear cell papillary RCC, or some low-grade mesothelial proliferation during routine histopathologic examination. PCE is characterized by cystic spaces with intracystic papillary projections lined by clear cells, very similar to clear cell carcinoma.^1^ Management consists of surgical excision of the tumor which is curative.

This case is of a 40-year-old man with a 1-cm right PCE and no history of VHLD. We describe the clinical presentation and gross and microscopic features associated with this entity.

## CASE REPORT

A 40-year-old man, non-smoker, presented to our institution due to microscopic hematuria identified on routine urine analyses of four years duration. Renal ultrasonography performed at another institution showed no abnormalities. The patient denied trauma to the testicular / perineal bump or history of infections including sexually transmitted diseases. The patient also denied having had any foley catheters or history of kidney stones. However, he did complain of incomplete bladder emptying, difficulty urinating, slow stream, and frequency. There was family history of renal cell carcinoma (cousin) and thyroid cancer (sister). Medical history was relevant for hypercholesterolemia for which the patient took atorvastatin 10mg once daily. The patient had a surgical hernia repair. He had no known allergies. He was sexually active and monogamous.

Vital signs on admission were as follows: blood pressure 133/85mmHg, pulse 84 beats per minute, respiratory rate 16 per minute, temperature 97.8^o^F, and SpO_2_ 98%. Body mass index (BMI) was 25.50 kg/m^2^ (height 1.829 meters and weight 85.3 kg). Physical examination revealed a circumcised penis, high-riding left testicle and a right epididymal cystic-like nodule.

**Figure 1:**
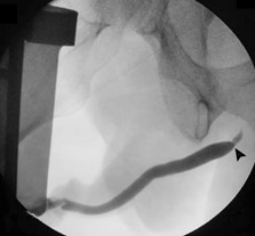
Retrograde urethrogram shows proximal short and narrow bulbar urethral stricture (arrowhead).

Retrograde urethrogram was performed by introducing contrast through a catheter-tip syringe into the urethra. Fluoroscopic images showed a proximal short and narrow bulbar urethral stricture (Figure 1), which explained patient's complaints of incomplete bladder emptying, difficulty urinating, slow stream, and frequency. Accordingly, cystoscopy with urethral balloon dilation was accomplished.

**Figure 2:**
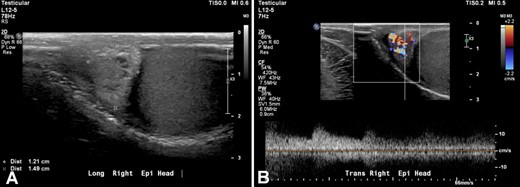
Color flow and spectral doppler of the right epididymis in sagittal plane. Results demonstrated: **A –** a right paratesticular (epididymal) mass (1.49 cm in greatest dimension); **B** – with increased vascularity.

Two months later, the patient continued to have mild urgency during the day and nocturia with not much improvement in his symptoms. He also complained of right back pain that radiated to the right testicle. Sitting down and laying down made it better, while standing made it worse. The pain was more prominent in the morning. The patient denied trauma to the genital area. He also started feeling a “liquid filled sac” on the right testicle. Testicular sonogram was performed revealing a 1.2 cm hypoechoic, hypervascular mass in the right epididymal head (Figure 2).

Serum LDH isoenzymes were within normal limits (LDH: 146 IU/L (Reference range (RR): 121 - 224 IU/L); LDH-1: 31% (RR: 17-32%); LDH-2: 35% (RR: 25-40%); LDH-3: 18% (17-27%); LDH-4: 7% (5-13%); LDH-5 9% (4-20%)). Alpha-Fetoprotein (AFP) was also normal at 0.9 ng/mL (RR: 0.0-8.0 ng/mL) and serum beta-HCG was negative. Differential diagnosis included focal epididymitis, adenomatoid tumor, lymphoma, or mesenchymal tumor. The supratesticular mass was excised and sent for pathologic evaluation.

**Figure 3:**
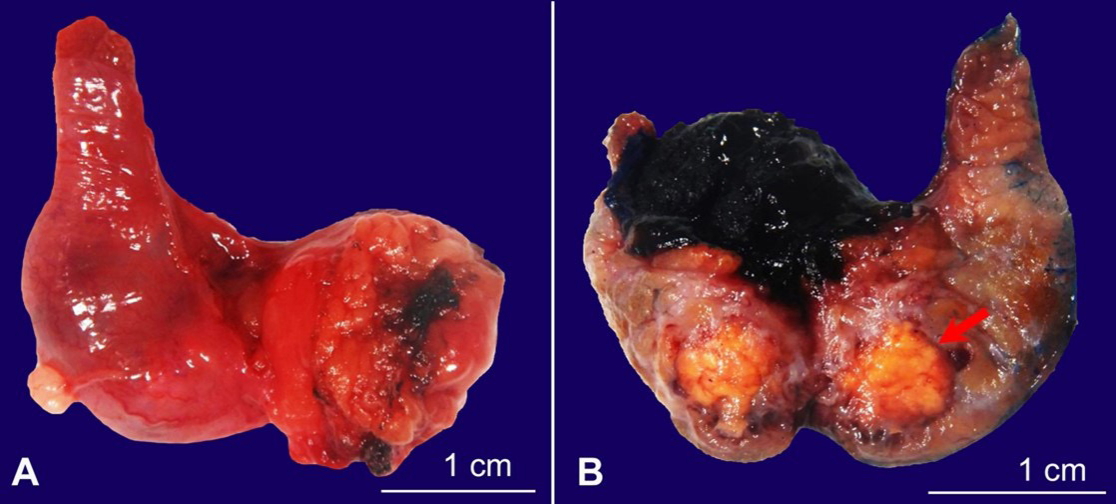
Gross examination of the resected right epididymis. **A –** Segment of epididymis with attached yellow, lobulated adipose tissue and tan, pink, smooth external surface. **B** – Cut section through the specimen revealed an irregular, bright yellow, solid mass that measured 1.1 x 0.8 x 0.8 cm, located at 0.2 cm from the resection margin.

Gross pathological examination demonstrated a 1.1 x 0.8 x 0.8 cm irregular, bright yellow, solid mass within the right epididymis (Figure 3).

**Figure 4:**
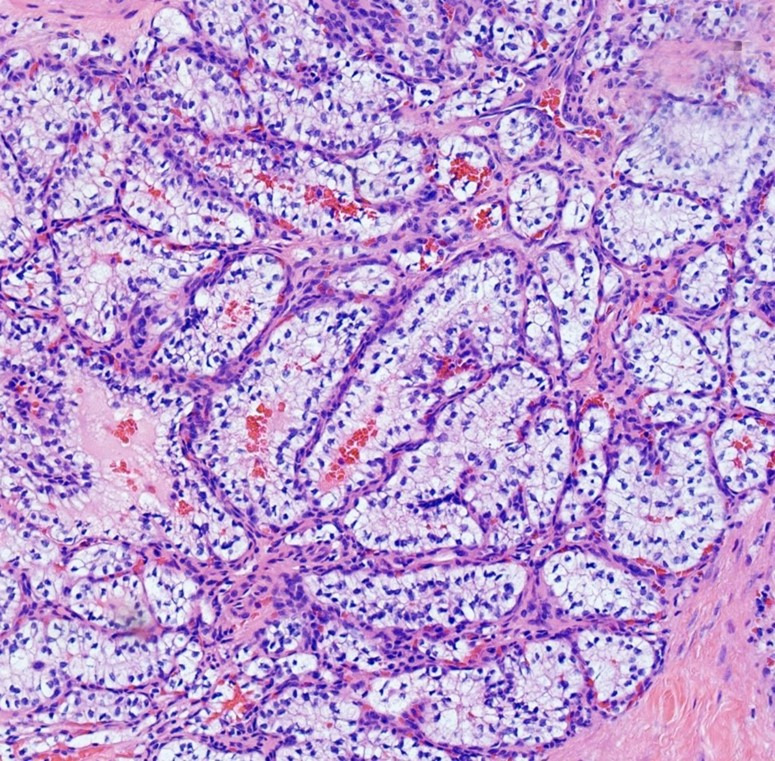
Photomicrographs of the epididymis showing epididymal mass with clear cell neoplasm and tubular/glandular morphology (H&E, 20x objective).

Microscopic examination revealed a clear cell neoplasm with tubular/glandular morphology (Figure 4). At this point, metastatic clear cell renal cell carcinoma (CCRCC) and clear cell papillary RCC were high in the differential diagnosis. However, the latter is extremely indolent with metastasis almost unheard of, and the patient's course of presentation with symptoms extending over a period of 4 years makes a malignant neoplasm less likely, favoring a benign entity.

**Figure 5:**
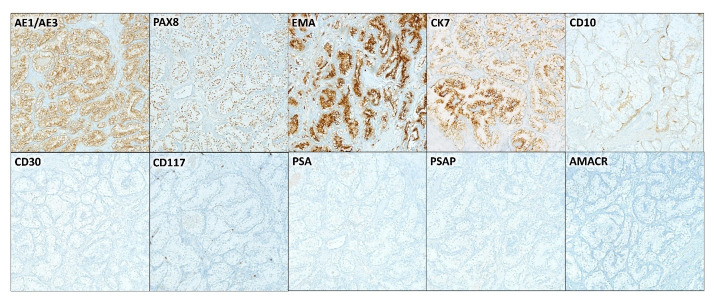
Photomicrographs of the epididymis examination using immunohistochemical stains. Tumor cells were positive for pankeratin (AE1/AE3), PAX8, EMA, and CK7. CD10 stain showed equivocal weak focal staining. PSA, PSAP, racemase (AMACR), CD30, and CD117 were all negative. Microscopic images were examined at 20x objective.

On immunohistochemical staining the tumor cells were positive for pankeratin (AE1/AE3), PAX8, cytokeratin 7 (CK7), and epithelial membrane antigen (EMA). A CD10 stain showed equivocal weak focal staining. The stains for prostate specific antigen (PSA), prostate-specific acid phosphatase (PSAP), racemase (AMACR), CD30, and CD117 were negative (Figure 5).

Taking into consideration that the epididymal mass was present on a CT scan from 2019 (performed at another institution), and that no renal masses were present (which make metastatic renal cell carcinoma an unlikely diagnosis), the histologic features and immunohistochemical findings were considered to represent a papillary cystadenoma of the epididymis (PCE).

Germline sequencing using a next-generation sequencing panel of 84 genes on the Invitae Multi-Cancer panel was performed (Supplement Material 1) to rule out Von Hippel-Lindau disease (VHLD).^34^ Results yielded heterozygous mutations in three genes - *CASR* (variant c.2417T>G (p.Phe806Cys)), *POT1* (variant c.526G>A (p.Gly176Arg)), and *RAD51D* (variant c.629C>A (p.Ala210Glu)), all three of which have not been reported in PCE before. *CASR* acts as a tumor suppressor or an oncogene, depending on the cancer site, and it is associated with an increased risk of colorectal, prostate, breast, and pancreatic cancers.^35^ *POT1* gene has been specifically reported in melanoma, chronic lymphocytic leukemia (CLL), angiosarcoma, and glioma.^28^ The *POT1* variant found in our case (variant c.526G>A (p.Gly176Arg)) has been linked to the hereditary cancer-predisposing syndrome. However, clinically it remains of uncertain significance due to the lack of studies tackling its role in human diseases.^36^ Similarly, the clinical significance of *CASR* (variant c.2417T>G (p.Phe806Cys)) and *RAD51D* (variant c.629C>A (p.Ala210Glu)) is yet to be determined.^37^ There have been reported associations of the pathogenic variants in *RAD51D* gene with tubo-ovarian carcinoma and breast cancer.^38^ At the 6-month follow-up, the patient was doing well and without symptoms.

## DISCUSSION

PCE is a benign tumor and the second most common neoplasm of the epididymis.^1^ It develops within the efferent ductules of the head of the epididymis as a solid, cystic, or partially cystic mass usually measuring up to 3 cm in diameter and is usually asymptomatic.^2^^,^^39^ This benign neoplasm can present in a unilateral or bilateral fashion, wherein bilateral lesions are more associated with von Hippel Lindau Disease (VHLD). It mostly affects young men, mean age 35 years, but has been reported between in patients as young as 16 and as old as 76 years of age.^1^ The modality of choice for evaluating paratesticular/epididymal masses is high-frequency ultrasonography and color flow doppler.^40^ Computed tomography (CT) is used for staging once a diagnosis of malignant neoplasia is made.^41^

PCE can mimic metastatic clear cell renal cell carcinoma (CCRCC), due to the histopathological features that these lesions share such as bright yellow cut surface and similar architecture, as well as clear “glycogen-rich” neoplastic cells in a highly vascularized background upon microscopic examination.^21^^,^^42^ Interestingly, patients with undiagnosed VHLD may have this as a primary presentation of their disease,^21^ prompting genetic testing in any patient with PCE, particularly those with bilateral lesions. This is particularly of utmost importance since PCE does not only resemble CCRCC histologically, but also because both are tumors observed in VHLD.^12^

Pathophysiologically, allelic loss of the *VHL* gene, located on the short arm of chromosome 13 (3p25-26), has been identified in all benign papillary tumors developing in VHL patients, including PCE.^2^^,^^21^ However, having a *VHL* mutation does not necessarily precede the diagnosis of papillary cystadenoma of the epididymis as there have been several cases reported where *VHL* mutations have been found in sporadic cases of PCE, which does not apply in our case.^12^ As mentioned previously, bilateral PCE is pathognomonic of VHLD.^42^ In a review of 59 cases of PCE, the latter was associated to VHLD in 29.3% of the cases.^26^ Two-thirds of the patients were also found to have VHLD with bilateral PCE.

In 1995, Gilcrease et al.^12^ pioneered the immunohistochemical characterization of multiple cases of PCE. Amongst these lesions, there was positive immunohistochemical staining for low and intermediate molecular weight keratins (CK7, CAM 5.2 and AE1/AE3), epithelial membrane antigen (EMA), and PAX2, many of which were positive in our case. Vimentin, alpha-1-antitrypsin, alpha-1-antichymotrypsin, S100, and carcinoembryonic antigen (CEA) can be variably positive and give discordant results.^1^ CD10 and CK20 stained negatively in previously reported cases.^1^^,^^8^^,^^24^ In our case, CK20 was negative whereas CD10 showed equivocal weak focal staining. Importantly, to differentiate PCE from CCRCC, useful stains which are strongly positive in CCRCC include CA-IX and CD10, both of which stain negatively with PCE. To the contrary, CK7 positivity supports a diagnosis of PCE and helps excludes metastatic CCRCC, as demonstrated in this report.^12^^,^^24^^,^^43^ Stains should be interpreted with caution as CK7 can be positive in papillary RCC and in cystic areas of CCRCC. In our case, CD10 and all prostate markers including PSA, PSAP, and AMACR stained negative excluding metastatic CCRCC and metastatic prostate cancer. It is noteworthy mentioning that PAX8 positivity is a diagnostic pitfall as many renal neoplasms stain positive for PAX8. Henceforth, it is always best to use more than one marker and to correlate with the morphology when making the diagnosis.

Management of PCE consists mainly of testicle-sparing surgical excision. In cases where local excision is not feasible, orchiectomy might be an option. Patients usually have an excellent prognosis after excision; recurrence of PCE was reported in only one case, which may have been due to incomplete initial excision.^1^ However, occurrence of cystadenocarcinoma was documented in two cases. Development of papillary cystadenocarcinoma, which is the malignant counterpart of PCE, is incredibly rare. In a study by Wang *et al*., this malignant entity had been reported in a 27-year-old man with right paraureteral metastases.^44^ Another case was also reported in a 43-year-old man with VHLD with testicular metastasis.^45^
